# Reducing the Environmental Impact of Health Care Conferences: A Study of Emissions and Practical Solutions

**DOI:** 10.1200/GO.23.00209

**Published:** 2024-02-15

**Authors:** Katie E. Lichter, Ali Sabbagh, Sasha Demeulenaere, Taylor Drew, Alexandra Conway, Leticia Nogueira, Gita Suneja, Kelsey Kirkwood, Karly Hampshire, Katherine Gundling, Arianne Teherani, Sapna E. Thottathil, Osama Mohamad

**Affiliations:** ^1^ University of California San Francisco, San Francisco, CA; ^2^ Stritch School of Medicine, Maywood, IL; ^3^ Geisel School of Medicine, Hanover, NH; ^4^ American Cancer Society, Kennesaw, GA; ^5^ University of Utah, Salt Lake City, UT; ^6^ American Society of Clinical Oncology, Alexandria, VA; ^7^ Columbia University, New York, NY; ^8^ MD Anderson Center, Houston, TX

## Abstract

**PURPOSE:**

We aimed to examine the impact of different conference formats (in-person, virtual, and hybrid) of the ASCO conference on greenhouse gas (GHG) emissions and to recommend sustainable options for future conferences.

**MATERIALS AND METHODS:**

This study used data on the number of attendees, their departure locations, and the type of attendance (in-person *v* virtual) provided by ASCO between 2019 and 2022. The GHG emissions resulting from air and ground travel, remote connectivity, conference space utilization, hotel stays, distributed conference materials, and electricity use were estimated for each year. Emissions were stratified by attendee country of origin, type of attendance, and year. Simulations were conducted to evaluate how changes in conference size, location, and format impact emissions, as well as estimate the resulting mitigations from adopting the proposed changes.

**RESULTS:**

The highest estimated GHG emissions, calculated in carbon dioxide equivalents (CO_2_e), were associated with the 2019 in-person conference (37,251 metric tons of CO_2_e). Although international attendees had the largest contribution to emissions in all years (>50%), location optimization models, which selected conference locations that most minimized GHG emissions, yielded only minimal reductions (approximately 3%). Simulations examining changes to the conference format, location, and attendance percentage suggested that hub-and-spoke, where multiple conference locations are selected by global region, or hybrid models, with both in-person and virtual components, are likely to cause the largest drops in emissions (up to 86%).

**CONCLUSION:**

Using historical conference data, this study identifies key aspects that can be modified to reduce emissions and consequently promote more sustainable and equitable conference attendance. Hybrid conferences may be the best solution to maintain the networking opportunities provided by conferences while balancing out their environmental footprint.

## Teaser

This study reveals hybrid oncology conferences slash emissions by 86%, advancing climate goals within oncology.

## INTRODUCTION

The current climate crisis poses an immediate and significant threat to human health, largely due to the release of greenhouse gas (GHG) emissions from human activities.^1,2^ Many industries, including health care and academia, are seeking innovative ways to reduce their climate impact. As air travel is the largest contributor to global GHG emissions from the transportation sector,^2^ reducing emissions from professional activities such as conference travel is critical. In fact, conference travel can account for up to 35% of a researcher's total carbon footprint, and a single conference can contribute up to 7% of a scientist's annual GHG emissions.^3^ Therefore, mitigating emissions from conferences at personal, institutional, and national levels is a crucial step for health care providers and the scientific community to prevent exacerbating poor patient and planetary health outcomes.

**CONTEXT**

**Key Objective**
What is the amount of carbon emissions associated with different conference attendance formats, and are there effective solutions to mitigate these emissions?
**Knowledge Generated**
In-person attendance has the highest associated amount of greenhouse gas emissions. Simulations conducted show that hub-and-spoke and hybrid attendance models can lead to considerable drops in emissions while maintaining the advantages of conference attendance and engagement.
**Relevance**
Our findings can be used by conference organizers and policymakers to plan inclusive and equitable conferences using an environmentally conscious approach.

The ASCO conference is a prominent event in the health care field held each year in Chicago, Illinois, attracting a large and diverse audience. In-person attendance at the 2019 conference drew over 33,000 attendees, whereas virtual attendance surged in 2020 and 2021, with over 41,000 and nearly 30,000 participants, respectively. In 2022, ASCO hosted a hybrid conference with both virtual and in-person attendance with nearly 45,000 attendees, 24,000 in-person and 10,000 virtual. Virtual conferencing has been shown to significantly reduce GHG emissions because of restricted participant travel, increase diversity among attendees, and reduce barriers to attendance, such as time, health, or financial constraints.^4-6^

Previous studies have focused on emissions caused by in-person conference-related travel and extrapolated potential benefits to virtual conferences^7-9^; however, there are limited quantitative assessments of the largest contributors of conference-related emissions and propose targeted and sustainable alternatives.^10-12^ Using attendance data from 2019 to 2022 ASCO conferences, this study addresses these gaps by conducting a quantitative analysis into the largest contributors of conference-related emissions, as well as present potential GHG emissions reductions associated with alternative, more sustainable models.

## MATERIALS AND METHODS

### Travel Distance Calculations

Data on the origin of US conference attendees by zip code, origin of international attendees by country, and participation format (in-person or virtual) from 2019 to 2022 were provided by ASCO staff. In accordance with the Common Rule, informed consent and study approval were not required since the data were deidentified.

For in-person attendance in 2019 and 2022, the conference was hosted in Chicago, Illinois. US attendees were matched to their nearest major airports using factors such as airport size, annual enplanements, and distance from the attendee's zip codes. Zip codes were validated and linked to their centroid location using 2021 zip code tabulation area data.^13^ We excluded US participants with zip codes that did not match the centroid data from the analysis (n = 3,288, <2%). We used spatial data from the US Department of Transportation together and passenger data from the Federal Aviation Administration to identify 240 US airports with ≥100,000 enplanements in 2019.^14-16^ We conducted a spatial join between zip code centroids represented in the ASCO attendee data and the 240 airports identified using ArcGIS Pro. We used Euclidean geometry to determine the airports that minimized the distance to each attendee's registered zip code.

For international attendees, we assumed they traveled from either a medium or large airport with the shortest total distance to all other airports in their country of origin and did not participate in the virtual component. Participants from countries with no medium or large airports were assumed to travel from small airports. Airport information by country was obtained using data from The World Bank Data Catalog.^17^ We excluded international participants from countries that lacked International Organization for Standardization-3,166 country codes, which were used to map ASCO data and airport database data (n = 3, <1%).

The distances between the matched airports of origin and Chicago O'Hare International Airport (ORD) were calculated using the Vincenty (ellipsoid) method.^18,19^ ORD was chosen as the assumed airport as it is the primary international hub for Chicago. The coordinates for all airports were obtained using an online database.^20^

### Emission Calculations

To estimate travel-related emissions, we made the following assumptions. Participants traveling <300 miles to the conference were assumed to have driven there.^21,22^ Although the ASCO conference runs for 10 hours per day for 5 days, attendees were assumed to attend the conference for 6 hours per day for 3 days. Participants traveling 40 miles or more stayed at a four-star hotel within walking distance. An additional 100 miles of driving to and from airports was assumed for attendees using air travel.^6,21,22^ We estimated GHG emissions in units of carbon dioxide equivalents (CO_2_e) for each in-person attendee, including emissions from travel, conference space, hotel stay, electricity, gas, and materials. CO_2_e travel consumption was estimated using the Environmental Protection Agency GHG Tools and the Department of Transportation Bureau of Transportation Statistics.^23,24^ CO_2_e consumption for hotel stay and conference space utilization (including electricity, gas, and materials) was estimated using the Hotel Footprinting Tool and MyClimate Carbon Offset calculator.^25,26^ We used the square footage of McCormick Place, the convention center in central Chicago where the ASCO is hosted, to estimate total space utilization in this calculation. We did not include food, food waste, local transportation, and other disposables in our calculations. For virtual attendees, emissions were estimated on the basis of CO_2_e from standard video platform use and associated electricity.^27^

### Simulations

To estimate the potential benefit of different conference models on CO_2_e emissions, we performed the simulations listed below. Note that simulations A, B, and C were specific to in-person conference formats and were only applied to data from 2019 to 2022. All simulations were calculated using R.

#### A. Removing Outliers

We estimated the decrease in total emissions associated with excluding travelers with extreme distances, defined as distances greater than two standard deviations from the average distance to the conference center.

#### B. Alternative Locations

This simulation estimated emissions associated with holding the conference at different locations, specifically Honolulu (Hawaii), Vienna (Austria), and New York City (New York), which tend to be popular oncology conference locations.

#### C. Minimizing Distances and Emissions

With flight-related emissions likely to make up a significant proportion of overall emissions, we conducted a simulation estimating the benefit of a conference model aimed at minimizing overall distance traveled and compared the results of this model to an optimal case scenario that minimizes overall emissions. Additionally, we performed an analysis where the conference location was selected to minimize CO_2_e emissions from US participants only (first simulation) and from all participants (second simulation).

#### D. Hybrid Model

This simulation is intended to quantify the expected benefit of hybrid conferences using worst case and best case analyses, where attendees from locations with the lowest and highest emissions, respectively, are assumed to have attended virtually. This approach allows us to obtain a range of emissions reduction associated with conferences held in a hybrid format.

#### E. Hub-and-Spoke Models

In this simulation, we divided countries into six subregions: North America, Europe, the Middle East and Africa, Latin and South America, and the Caribbean, Asia, and Oceania. We selected a hub within each region. Attendees in countries (spokes) from that subregion were assumed to travel to the hub (also a country in the subregion) to attend the conference in-person. In the first simulation, we selected the country that minimized the distance traveled from all countries in the subregion as the hub. For the second simulation, we chose more realistic hub locations where conferences were more likely to be held (ie, locations that already host major conferences). For example, while Sudan is chosen as the hub for the Middle East and North Africa in the ideal scenario of the simulation because of it minimizing travel distance for attendees in the region, Egypt was selected as the hub in the realistic scenario of the simulation.

#### F. Turning Cameras Off

Research has demonstrated that turning cameras off in virtual sessions can significantly reduce CO_2_e emissions, up to 96%.^27^ Thus, we assumed that all attendees turned off their cameras and conducted the simulation, accordingly, resulting in emissions that were only 4% of standard virtual emissions.

### Statistical Analysis

All calculations and simulations were performed using R statistical software (v.4.1.2, R Foundation, Vienna, Austria), and *P* values <.05 were considered statistically significant. The details on the assessment of statistical significance can be found in the Data Supplement.

## RESULTS

### Conference Emissions and Attendees

**FIG 1:**
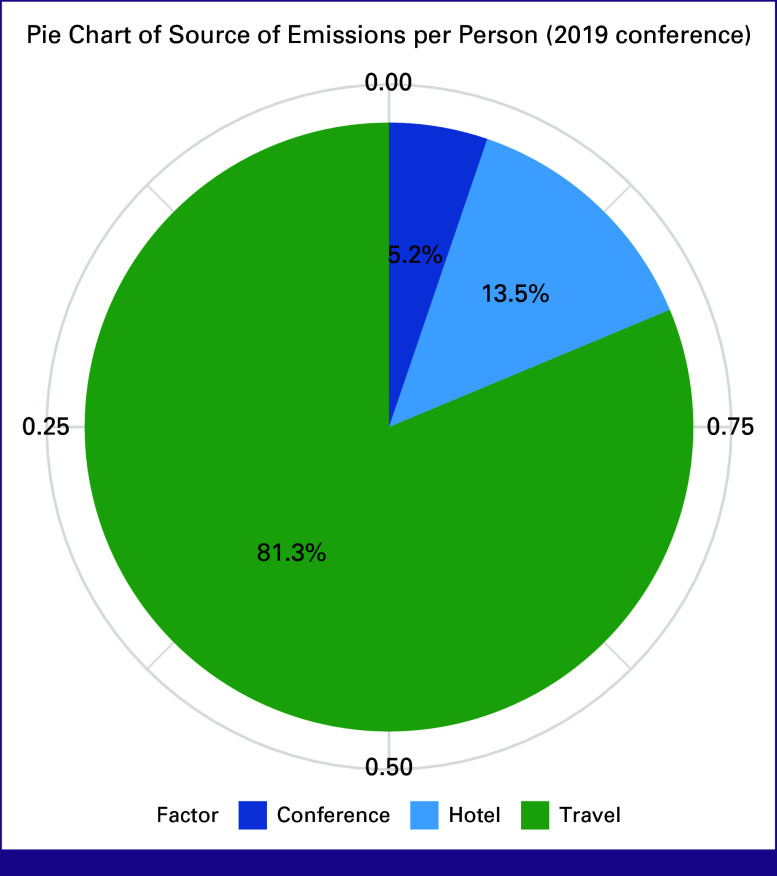
Pie chart of source of emissions per person (2019 conference).

Attendance and emissions from 2019 through 2022 ASCO conferences are summarized in Table 1. CO_2_e emissions were the highest in 2019 when attendance was fully in-person and the lowest in 2020 and 2021 when the conference was fully virtual. The total CO_2_e emissions for the 2019 in-person conference were 37,251.45 metric tons, which is equivalent to the annual emissions of 8,018 gasoline-powered passenger vehicles. This estimation assumes that an average US vehicle travels 11,500 miles per year with a fuel economy of 22.0 miles per gallon.^23^ Using the 2019 attendance data, we further stratified average emission contributions per person by travel, conference space utilization, and hotel stay (Fig 1). As expected, travel accounted for the majority of emissions per person (81.3%). By contrast, the average CO_2_e emissions for the virtual conference format were 99.17 metric tons, equivalent to the annual emissions of 21.4 gasoline-powered passenger vehicles. The 2022 hybrid conference resulted in 20,190 metric tons of CO_2_e emissions, equivalent to the annual emissions of 4,351 gasoline-powered passenger vehicles. The adoption of a hybrid format led to a significant drop in emissions/per person compared with the 2019 conference (*P* < .001). The calculated average per-person CO_2_e emissions for the in-person, virtual, and hybrid formats were 1.11, 0.0028 (difference with the emissions from the 2019 conference is 1.1071, *P* < .001, 95% CI (1.0979 to 1.1163), and 0.5747 metric tons (difference with the emissions from the 2019 conference is 0.5352, *P* < .001 [95% CI, 0.5236 to 0.5469]), respectively.

**TABLE 1:** Attendance and Emissions of the ASCO Conferences Between 2019 and 2022

Attendance and Emission	Y2019	Y2020	Y2021	Y2022
Attendance				
Total No. of attendees (No.)	33,562	41,199	29,634	35,133
Total No. of omitted attendees (No.)	829	1,070	556	833
No. of in-person attendees, percentage out of total, No. (%)	33,562 (100.00)	0 (0.00)	0 (0.00)	24,360 (69.34)
No. of virtual attendees, percentage out of total, No. (%)	0 (0.00)	41,199 (100.00)	29,634 (100.00)	10,773 (30.66)
No. of US in-person attendees, percentage out of total, No. (%)	18,731 (55.81)	0 (0.00)	0 (0.00)	17,295 (49.23)
No. of US virtual attendees, percentage out of total, No. (%)	0 (0.00)	19,241 (46.70)	13,597 (45.88)	3,594 (10.23)
No. of international in-person attendees, percentage out of total, No. (%)	14,831 (44.19)	0 (0.00)	0 (0.00)	7,065 (20.11)
No. of international virtual attendees, percentage out of total, No. (%)	0 (0.00)	21,958 (53.30)	16,037 (54.12)	7,179 (20.43)
No. of percentage of attendees traveling <300 miles, No. (%)	2,330 (6.94)	0 (0.00)	0 (0.00)	2,126 (6.05)
No. of percentage of attendees traveling 300-3,000 miles, No. (%)	17,767 (52.94)	0 (0.00)	0 (0.00)	16,161 (46.00)
No. of percentage of attendees traveling >3,000 miles, No. (%)	13,465 (40.12)	0 (0.00)	0 (0.00)	6,073 (17.29)
Emissions				
Total CO_2_e emissions (metric tons, t)	37,251.45	115.36	82.98	20,190.01
Total CO_2_e emissions (t) ppa	1.11	0.0030	0.0030	0.58
Total CO_2_e emissions (t) from in-person attendance	37,251.45	0.00	0.00	20,159.84
Percentage CO_2_e emissions (%) from in-person attendance	100.00	0.00	0.00	99.85
Total CO_2_e emissions (t) from in-person attendance ppa	1.11	0.00	0.00	0.83
Total CO_2_e emissions (t) from attendees traveling <300 miles	362.23	0.00	0.00	339.39
Percentage CO_2_e emissions (%) from attendees traveling <300 miles	0.97	0.00	0.00	1.68
Total CO_2_e emissions (t) from attendees traveling 300-3,000 miles	9,110.68	0.00	0.00	8,343.25
Percentage CO_2_e emissions (%) from attendees traveling 300-3,000 miles	24.46	0.00	0.00	41.32
Total CO_2_e emissions (t) from attendees traveling >3,000 miles	27,778.55	0.00	0.00	11,477.21
Percentage CO_2_e emissions (%) from attendees traveling >3,000 miles	74.57	0.00	0.00	56.85
Total CO_2_e emissions (t) from US attendees	8,657.95	53.87	38.07	8,076.1
Percentage CO_2_e emissions (%) from US attendees	23.24	46.70	45.88	40.00
Total CO_2_e emissions (t) from international attendees	28,593.51	61.48	44.9	12,113.90
Percentage CO_2_e emissions (%) from international attendees	76.76	53.30	54.12	60.00
Total CO_2_e emissions (t) from virtual attendance	0.00	115.36	82.98	30.16
Percentage CO_2_e emissions (%) from virtual attendance	0.00	100.00	100.00	0.15
Total CO_2_e emissions (t) from virtual attendance ppa	0.00	0.0027	0.0028	0.0028
Total CO_2_e emissions greenhouse equivalence (gasoline-powered passenger vehicles driven for a year)	8,009.06	24.80	17.84	4,340.85
Total in-person attendance CO_2_e emissions greenhouse equivalence	0.24	0.00	0.00	4,334.37
Total virtual attendance CO_2_e emissions greenhouse equivalence	0.00	0.00060	0.00060	6.48

Abbreviations: CO_2_e, carbon dioxide equivalents; ppa, per person average.

**FIG 2:**
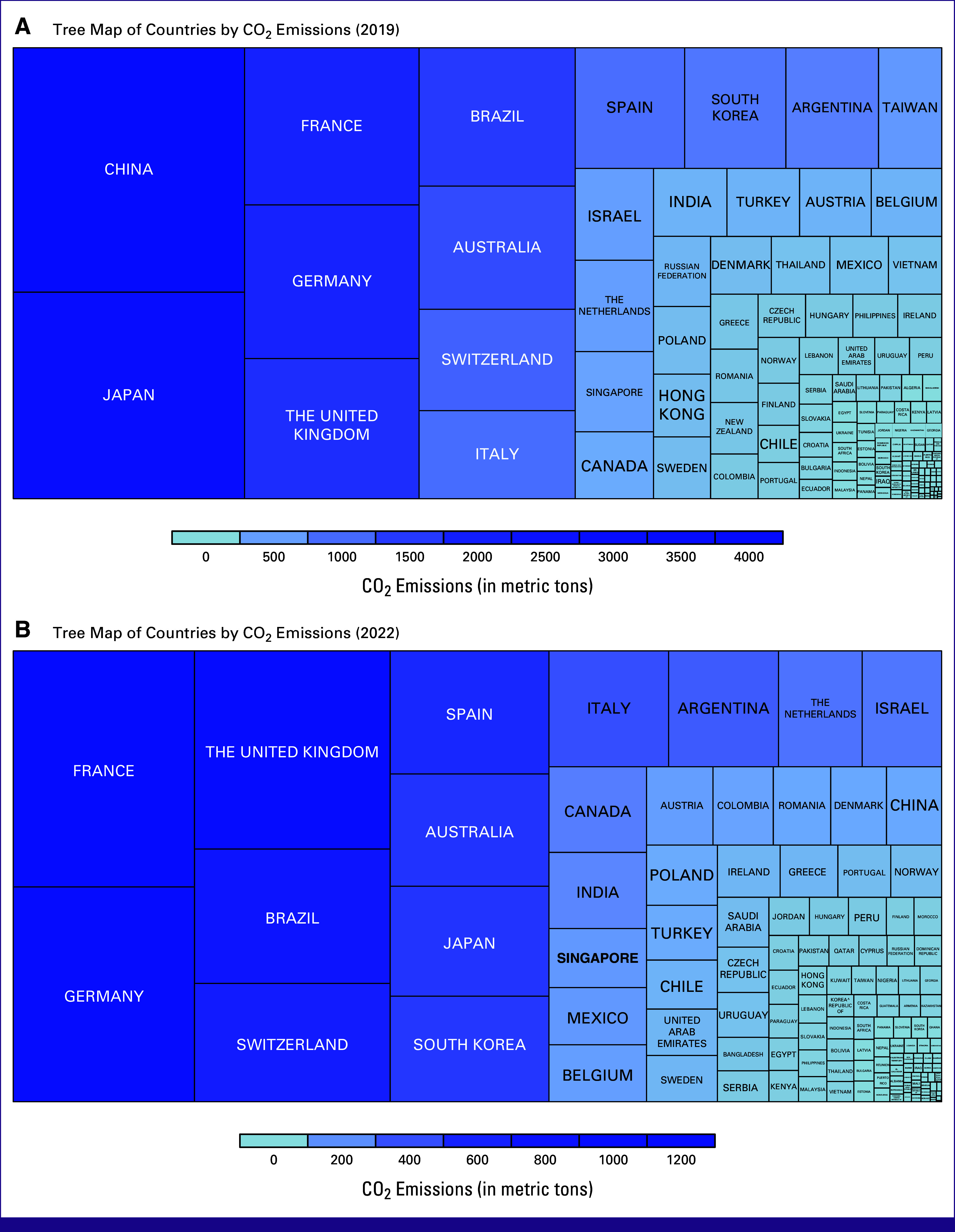
Emissions per person (in metric tons of CO_2_e) from international attendance by country in (A) 2019 and (B) 2022 in person conferences, and tree maps showing emissions by country (in metric tons of CO_2_e) for (A) 2019 and (B) 2022 conferences. CO_2_e, carbon dioxide equivalents.

The implementation of the fully virtual format in 2020 resulted in a 22.75% increase in attendance compared with the 2019 in-person conference (Table 1). This trend did not continue in 2021, where attendance decreased compared with the 2019 conference. The 2021 conference, which was fully virtual, had the lowest emissions because of the conference modality and the lowest attendance of the 4 years. Furthermore, the percentage of international attendees was higher in 2020 and 2021, which is likely attributed to the convenience of virtual formatting. International attendees were the largest contributors to CO_2_e emissions, regardless of whether the conference was in-person or virtual, with international attendees accounting for more than 50% of total CO_2_e emissions. Reduced emissions from international attendees were observed between 2019 and 2022, decreasing from approximately 29,000 to 12,000 metric tons of CO_2_e. This is attributed to the adoption of a hybrid conference model and to travel restrictions that were imposed in response to the COVID-19 pandemic. Figure 2 illustrates the emissions per person from international attendees in 2019 and 2022. In 2022, emissions per person from most countries were found to be <800 metric tons of CO_2_e, which is well below 2019 levels. Notably, the emissions from China were nearly nonexistent in 2022, which could be attributed to COVID-19–related travel restrictions.

### Simulations

Table 2 summarizes the emissions of different simulations of the ASCO conferences between 2019 and 2022. Simulation A shows that removing attendees from outlier locations has a modest effect on overall emissions, with reductions in overall emissions of approximately 7% and 4% for the 2019 and 2022 conferences, respectively. Although this translates to a statistically significant difference in emissions per person (*P* < .001), this difference remains minimal at an estimated 5% (95% CI, 0.040 to 0.066) decrease for the 2019 conference.

**TABLE 2:** Emissions of Difference Simulations of the ASCO Conferences Between 2019 and 2022

Simulation	Y2019	Y2020	Y2021	Y2022
Simulation A				
Total CO_2_e emissions (t) after removing outliers	34,653.40	NA	NA	19,391.79
Simulation B				
Total CO_2_e emissions (t) from in-person in Honolulu, Hawaii	66,899.56	NA	NA	46,307.28
Total CO_2_e emissions (t) from in-person in Vienna, Austria	53,518.96	NA	NA	39,885.51
Total CO_2_e emissions (t) from in-person in New York, New York	36,193.78	NA	NA	19,360.24
Simulation C				
Total CO_2_e emissions (t) from in-person minimizing all attendee travel distances	36,963.04	NA	NA	NA
Total CO_2_e emissions (t) from in-person minimizing US attendee travel distances	38,526.85	NA	NA	NA
Total CO_2_e emissions (t) from in-person minimizing US attendee travel distances	91,25.90	NA	NA	NA
Total CO_2_e emissions (t) from in-person minimizing US emissions	83,75.128	NA	NA	NA
Total CO_2_e emissions (t) from in-person minimizing all attendee emissions	36,139.10	NA	NA	NA
Simulation D				
Total CO_2_e emissions (t) from 100% in-person conference	37,251.45	51,658.41	36,260.08	36,544.18
Total CO_2_e emissions (t) from hybrid model: 25% virtual, 75% in-person (bc)	21,110.96	30,301.95	22,330.98	21,296.61
Total CO_2_e emissions (t) from hybrid model: 50% virtual, 50% in-person (bc)	12,716.86	18,402.30	12,353.89	13,523.34
Total CO_2_e emissions (t) from hybrid model: 75% virtual, 25% in-person (bc)	5,001.55	6,884.66	5,045.32	5,143.58
Total CO_2_e emissions (t) from hybrid model: 25% virtual, 75% in-person (wc)	32,342.02	44,887.42	31,295.59	31,498.60
Total CO_2_e emissions (t) from hybrid model: 50% virtual, 50% in-person (wc)	24,628.56	33,369.84	23,989.16	23,118.82
Total CO_2_e emissions (t) from hybrid model: 75% virtual, 25% in-person (wc)	16,233.69	21,470.19	14,009.96	15,344.22
Total CO_2_e emissions (t) from 100% virtual conference	93.973	115.357	82.97	98.37
Simulation E				
Total CO_2_e emissions (t) from ideal hub-and-spoke model (six locations)	16,958.76	21,731.68	15,557.15	12,171.32
Total CO_2_e emissions (t) from realistic hub-and-spoke model (six locations)	15,142.64	19,433.25	14,248.91	16,549.01
Simulation F				
Total CO_2_e emissions (t) from virtual attendance with all cameras turned off	3.76	4.61	3.319	1.21

Abbreviations: bc, best case; CO_2_e, carbon dioxide equivalents; NA, not applicable; wc, worst case.

**FIG 3:**
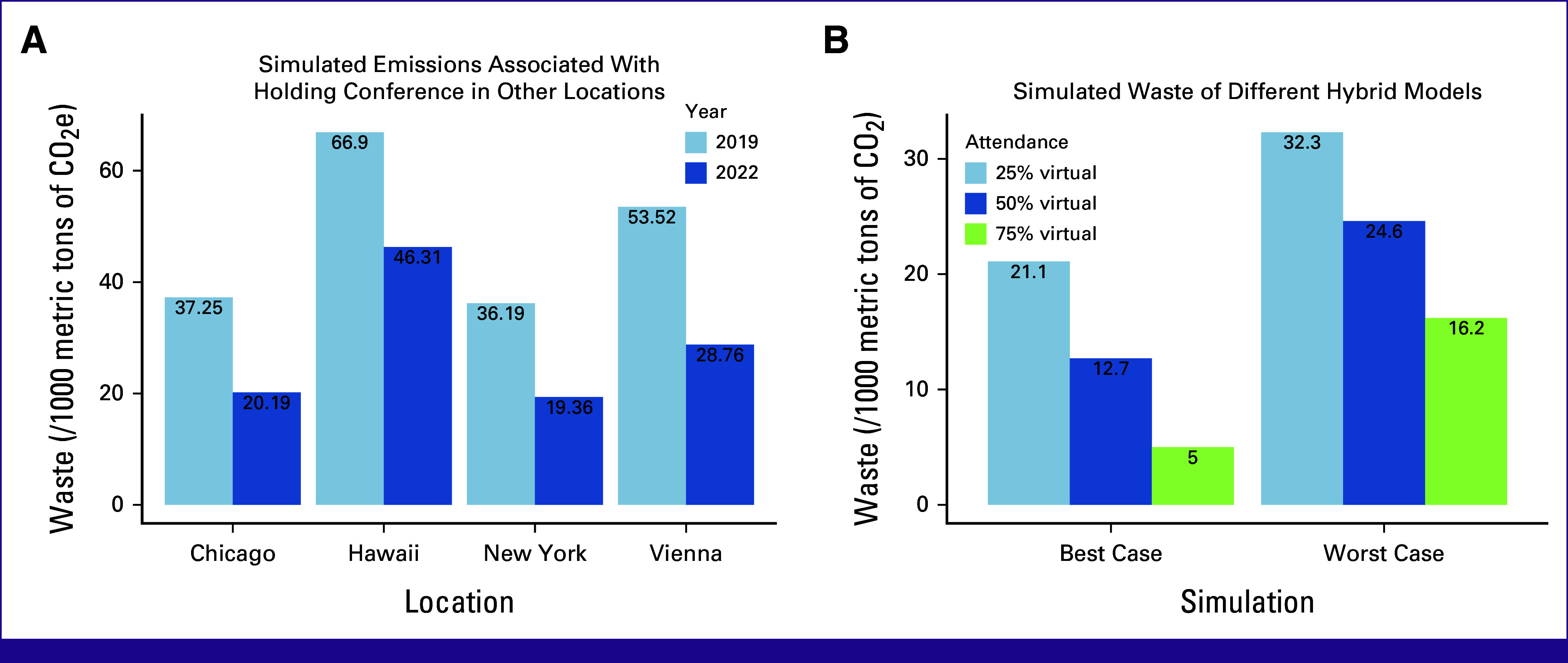
(A) Simulated emissions/person associated with holding the ASCO conference in alternative locations. (B) Graphs stratifying CO_2_e emissions based on the percentage of in person versus virtual attendees. CO_2_e, carbon dioxide equivalents.

The results of simulations B, C, and E show the contribution of conference location on overall emissions. Changing the location to other popular destinations (simulation B, Table 2 and Fig 3A) leads to considerable changes in emissions for the 2019 conference. Moving the conference to New York leads to approximately 2.8% decrease in overall emissions, whereas moving the conference to Vienna or Honolulu leads to approximately 44% and 80% increases, respectively. A similar trend is seen for the 2022 conference.

Simulation C shows the effect of a data-driven choice of the conference location. Selecting a conference center that minimizes distance traveled leads to an estimated approximately 0.77% drop in emissions. A more thorough analysis showed that the maximum drop in emissions that can be achieved on the basis of the choice of conference location alone amounts to approximately 2.99% reduction. The discrepancy between these two optimization scenarios is due to the contribution of the number of attendees to the calculation of emissions. While distance is an important factor, areas with a larger number of attendees can lead to higher total emissions than those with a lower number.

Simulation D (Table 2 and Fig 3B) highlights the potential reduction in emissions obtained by modifying the percentage of virtual attendance in a hybrid conference on the basis of the 2019 attendance mix. Even in the worst case, holding a conference with as little as 25% virtual attendance leads to approximately 13% drop in emissions for the 2019 conference. This reduction can go up to approximately 87% in the best case scenario, with 75% virtual attendance.

**FIG 4:**
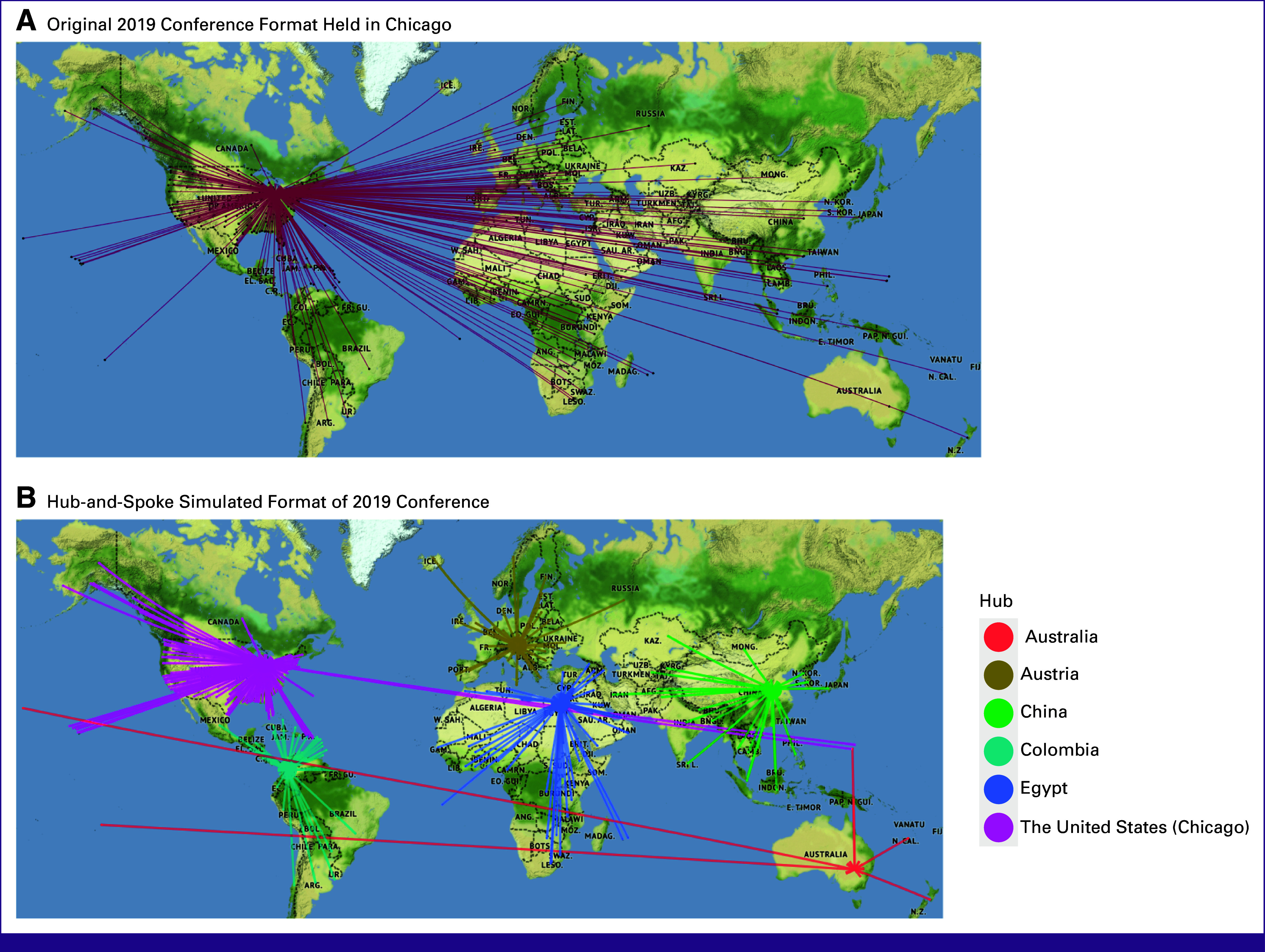
Travel patterns of participants attending the 2019 conference using (A) actual conference format and (B) hub-and-spoke format.

Simulation E shows the associated emissions reduction of a hub-and-spoke model, that is, having multiple conference locations with attendees allocated to their closest location (Table 2 and Fig 4). A hub-and-spoke model for the 2019 conference, for example, can reduce emissions by 54%-59%, on the basis of a choice of realistic and ideal locations, respectively.

## DISCUSSION

Preventing irreversible climate change will require significant emission reduction across all sectors by 2030 and achieving net-zero emissions by 2050.^1,27,28^ However, there has been little focus on addressing emissions from conference travel to meet these targets.^28-32^ Our study shows that although international attendance makes up most conference-related emissions (>50%), international attendance need not be limited to mitigate emissions. In fact, our simulations show that considerable emissions reduction can be achieved by restructuring conference parameters. This is evident in simulation D, where some level of virtual attendance resulted in a considerable decrease in conference-related emissions. Virtual conferencing has been criticized for compromising networking, professional development, and social interaction. However, studies have shown that virtual conferencing can provide structured networking opportunities while achieving workshop objectives comparable with in-person formats.^33^ Additionally, up to 60% of participants are willing to accept the downsides of virtual conferencing in exchange for personal and environmental benefits.^34^ Previous qualitative assessments of virtual and hybrid professional development conferences have identified flexibility, accessibility, and useful incorporation of technology as major assets of a virtual conference format that can help offset other downsides.^35^ Hybrid or virtual formats also have the potential to improve diversity, equity, and inclusion within the medical field by removing financial, physical, and temporal barriers for individuals with disabilities, caretaking responsibilities, and financial constraints.^4,34^ Although hybrid and virtual formats have already been successfully used in several fields, including surgery, dermatovenereology, and rheumatology, they have unexplored potential for further optimization and utilization.^36-38^ It is important to consider digital inequalities and ensure that virtual conferencing opportunities are inclusive and accessible to all including those with limited access to technology, internet, and/or electricity.

An intuitive solution would be to minimize the distances traveled by participants. However, as seen in the results of our simulations, this had only a small effect on overall emissions. Interestingly, selecting a conference location that minimizes emissions, even when in possession of full attendance information, leads to a minimal decrease (approximately 2.99%). This reduction floor can be overcome using a hub-and-spoke model. From our results, a hub-and-spoke model eliminates more than half of emissions while maintaining in-person attendance. However, although it successfully balances these desirable characteristics, it is not without drawbacks. A considerable proportion of health care research is published in the global north and such models might inadvertently exclude participants from the global south from important conversations and opportunities. Additionally, the extra planning required for multiple conference hubs may not be feasible for organizations at present; however, the utilization of novel technologies may soon mitigate this barrier. Successful hub-and-spoke conferences from other industries have used technology such as Mediasite, Barco, and Black Box, and growing interest in virtual conference alternatives will not only improve our current technology but drive further innovation.^39^ Future investigations should explore the incorporation of incentives, such as reduced conference fees, extended speaking sessions, and enhanced networking opportunities for virtual attendees, as these measures can promote equity of access and make virtual participation more appealing, encouraging a wider and more inclusive engagement in sustainability initiatives.

Conference organizers and attendees can also take immediate steps to incorporate environmental sustainability into their events. These steps can include reducing waste by swapping single-use materials for reusable or digital materials, implementing recycling and composting systems at all conferences, and using energy-efficient protocols.^40^ Providing environmentally responsible meal options, such as plant-based or locally sourced meals, and green transportation options can also help reduce emissions. Carbon offset programs can be used as an intermediary option until more robust and sustainable alternatives are available. By taking these actions, health care and academic conferences can lead by example in reducing GHG emissions and promoting sustainable practices. Figure 5 includes our previous work on impactful initiatives for conference organizers to significantly reduce emissions. Additionally, our GreenHealth Lab^41^ is launching a calculator, Network Greener, which will allow individuals and conference organizations to calculate emissions associated with their conference plans and provide information on how to offset these with both lifestyle and systemic changes. This tool also allows attendees to appropriately weigh the advantages of attending in person with the GHG emissions associated with attendance.

FIG 5(A) Commitment to Sustainability at Conferences for Individual Attendees and (B) Commitment to Sustainability at Conferences for Conference Organizers. Abbreviation: FSC, Forest Stewardship Council; HVAC, heating, ventilation, and air conditioning.

**Figure:**
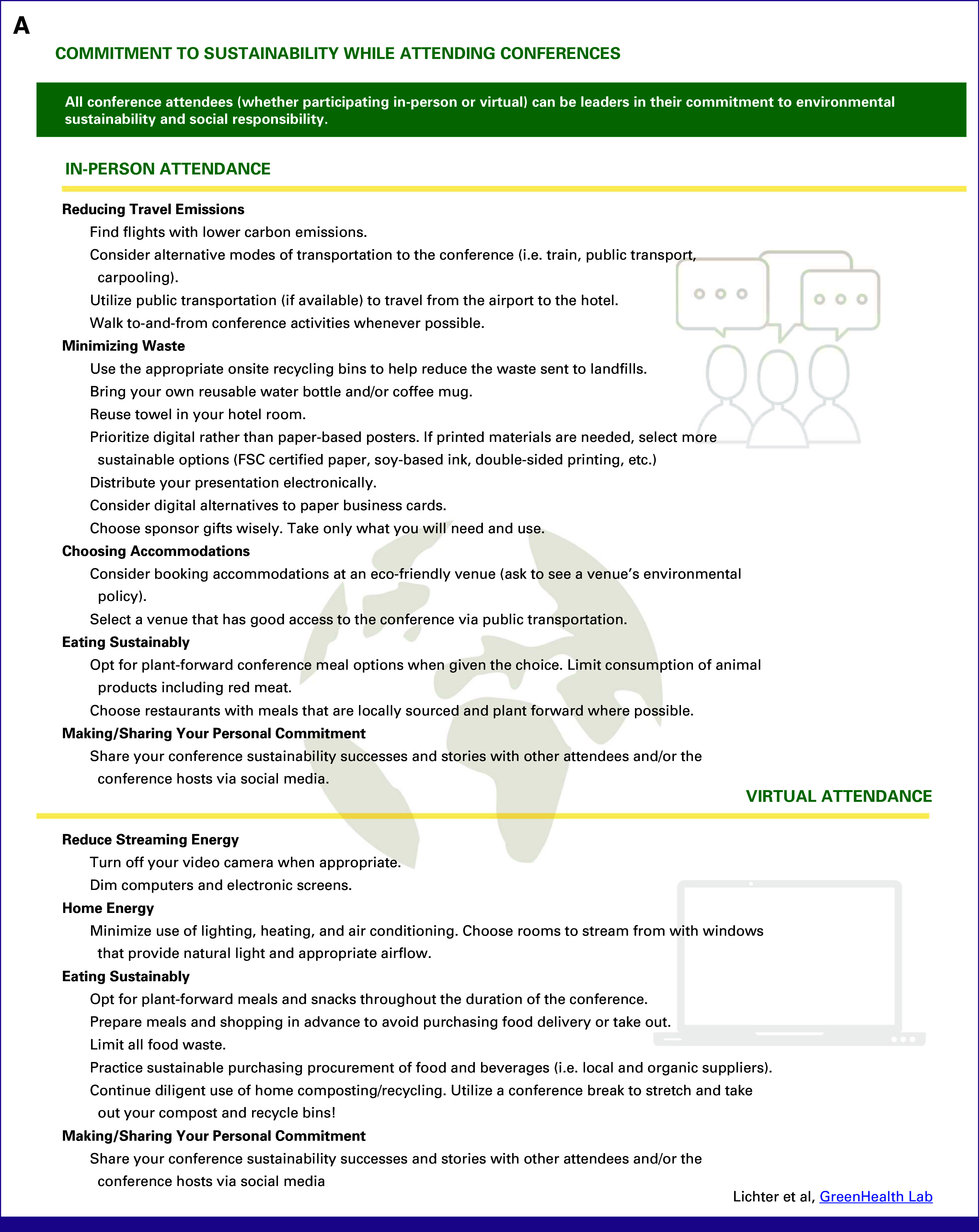

**Figure:**
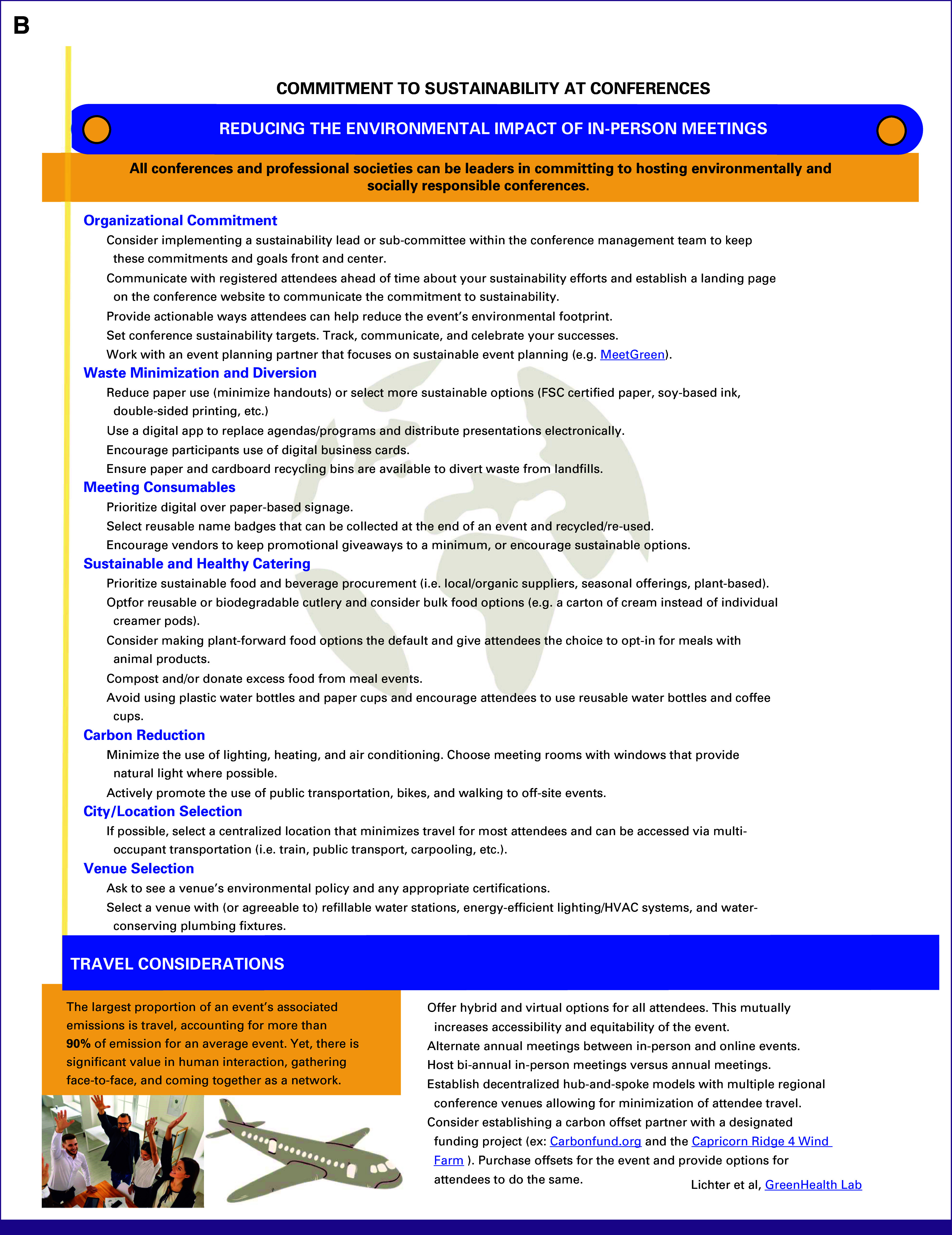

Our study offers valuable insights into the GHG emissions associated with health care conferences, but it is important to acknowledge its limitations. First, participants may have been misclassified to the airport of origin from their institutional zip code, although we expected this to even out across the population and have a negligible impact. Second, our calculations assumed that participants would miss some conference programming and not attend all events, which may underestimate emissions as full conference attendance would result in higher emissions. Additionally, COVID-19–related travel restrictions may have confounded our results, particularly in the decreased emissions observed with the 2022 hybrid model. Finally, our study did not account for factors such as layovers, waste disposal, electrical grid efficiency, alternative transportation, and food, which may increase emissions. The decision to exclude these factors was based on limited scientific literature and verified metrics. Future studies that include these factors are essential to gain a more comprehensive understanding of conference-related emissions. For example, although we could not quantify the emissions of single-use conference materials because of variability in types, composition, and quantity, it is a known source of waste and emissions that could be targeted with further research. Despite these limitations, our study offers a novel perspective on conference-related emissions which has been lacking in previous literature because of survey selection bias, smaller data sets, or failure to consider hybrid formats and emissions from virtual participation.^42-46^ By using actual data from the 2019 to 2022 ASCO conferences and modeling multiple alternative formats, including virtual format emissions, our study provides a pilot examination of conference-related emissions.

As health care providers, we bear a unique responsibility to reduce GHG emissions, especially considering the intrinsic emissions in health care activities that are difficult to minimize. Therefore, it is crucial to plan inclusive and equitable conferences that account for planetary harm. Our study provides quantitative evidence that virtual and hybrid conferencing significantly reduces GHG emissions, which can encourage more intentional and conscientious planning of future health care conferences. Utilization of environmentally conscious strategies can foster greater participant inclusion and reduce GHG emissions.
